# Fluorescent labeling in semi-solid medium for selection of mammalian cells secreting high-levels of recombinant proteins

**DOI:** 10.1186/1472-6750-9-42

**Published:** 2009-05-11

**Authors:** Antoine W Caron, Claire Nicolas, Bruno Gaillet, Ismaïla Ba, Maxime Pinard, Alain Garnier, Bernard Massie, Rénald Gilbert

**Affiliations:** 1 Institut de Recherche en Biotechnologie, Conseil National de Recherches du Canada, 6100 Royalmount Avenue, Montréal, QC H4P 2R2, Canada; 2 Chemical Engineering Department, Université Laval, Québec, QC, G1K 7P4, Canada; 3 Département de Microbiologie et Immunologie, Faculté de Médecine, Université de Montréal, Montreal, QC, H3C 3J7, Canada; 4 INRS-IAF, Université du Québec, Laval, QC, H7N 4Z3, Canada; 5 Neuromuscular research group, Montreal Neurological Institute, Montreal, QC, H3A 2B4, Canada

## Abstract

**Background:**

Despite the powerful impact in recent years of gene expression markers like the green fluorescent protein (GFP) to link the expression of recombinant protein for selection of high producers, there is a strong incentive to develop rapid and efficient methods for isolating mammalian cell clones secreting high levels of marker-free recombinant proteins. Recently, a method combining cell colony growth in methylcellulose-based medium with detection by a fluorescently labeled secondary antibody or antigen has shown promise for the selection of Chinese Hamster Ovary (CHO) cell lines secreting recombinant antibodies. Here we report an extension of this method referred to as fluorescent labeling in semi-solid medium (FLSSM) to detect recombinant proteins significantly smaller than antibodies, such as IGF-E5, a 25 kDa insulin-like growth factor derivative.

**Results:**

CHO cell clones, expressing 300 μg/ml IGF-E5 in batch culture, were isolated more easily and quickly compared to the classic limiting dilution method. The intensity of the detected fluorescent signal was found to be proportional to the amount of IGF-E5 secreted, thus allowing the highest producers in the population to be identified and picked. CHO clones producing up to 9.5 μg/ml of Tissue-Plasminogen Activator (tPA, 67 kDa) were also generated using FLSSM. In addition, IGF-E5 high-producers were isolated from 293SF transfectants, showing that cell selection in semi-solid medium is not limited to CHO and lymphoid cells. The best positive clones were collected with a micromanipulator as well as with an automated colony picker, thus demonstrating the method's high throughput potential.

**Conclusion:**

FLSSM allows rapid visualization of the high secretors from transfected pools prior to picking, thus eliminating the tedious task of screening a high number of cell isolates. Because of its rapidity and its simplicity, FLSSM is a versatile method for the screening of high producers for research and industry.

## Background

Chinese Hamster Ovary (CHO) cells continue to be a choice mammalian system for producing recombinant biopharmaceuticals despite relatively low yields and high costs. This is largely due to their ability to properly fold and post-translationally modify complex recombinant proteins such as antibodies for human administration [1-3]. Advances in cell culture technologies, bioprocesses and metabolic engineering have remarkably increased yields of recombinant proteins produced by mammalian cells [1,2,4]. Stable cell lines, employed for recombinant protein production, are usually obtained by isolating and characterizing high producers after transfection and random integration of the gene of interest. Since the proportion of high producers in the transfected cell pool is extremely small, several methods have been designed to facilitate their screening and selection [5].

"Blind" selection of high producers using limiting cell dilution in 96 well plates after transfection, is a common method. Following the formation of drug resistant colonies, blind cloning requires amplifying and individually testing hundreds (sometimes thousands) of these drug resistant colonies or cell populations, most of which will eventually be discarded as non/low producers. Although the technique is well established and reliable, it is a labor-intensive, time-consuming process. The advent of natural, low toxicity fluorescent markers such as the Green Fluorescent Protein (GFP) family opened a new era for reporter genes and detection of recombinant protein expression. Fluorescent markers are used in fusion proteins, as co-expression indicators, or in dicistronic configuration to help visualize cells expressing the proteins of interest within a mixed population [6-8]. High producers can be isolated by using microscopy in conjunction with micromanipulators [9], or with automated cell pickers, as well as by fluorescence activated cell sorting (FACS) [7,10]. Unfortunately, linking the production of a protein of interest to expression of fluorescent markers means that GFP or similar reporters will be present in the biomass/culture medium, which is largely undesirable in the context of biopharmaceuticals destined for human administration. GFP can be replaced with a cell surface marker normally absent in CHO cells, such as CD20, in order to isolate high producers by FACS using a fluorescent antibody against the marker [11].

A different approach is to attempt at labeling the secreted product itself, the main difficulty being to retain the product around the cells in order to identify high producers. This strategy can be traced back to the selection process for myeloma mutants which involved plating the cells in semi-solid medium containing agarose [12]. Their method allowed not only immobilization of the cells to form distinct clonal colonies but it also retained the secreted antibodies in the immediate vicinity of cells. When an anti-immunoglobulin overlay was added to the semi-solid medium, a precipitate formed only around positive colonies secreting antibodies. The method was used extensively for hybridoma selection and eventually, methylcellulose was found to be superior to agarose for mammalian cell growth [13]. Over the last decade, the use of single-cell gel matrices containing specific labels, has been combined with flow cytometry to detect and sort high secretors. Molten biotinylated agarose is used in conjunction with avidin and a biotinylated capture antibody against the recombinant protein [14]. Labeled cells are sorted by flow cytometry once the cell suspension are emulsified into small droplets. Cells can also be labeled with biotin and conjugated with a capture antibody. The accumulated recombinant protein is then visualized using a second fluorescent antibody [15,16]. A disadvantage of these methods is their complexity since a number of capture and labeling molecules are needed and that optimization of the labeling or growth condition may be necessary. A variation of this approach is to coat the plates directly with the capture molecule [17]. Recently, a method combining cell colony growth in methylcellulose-based medium with detection by unlabeled [18] or fluorescently labeled secondary antibody or antigen [19] has shown promise for the selection of cell lines secreting recombinant antibodies. It is not clear however if such method, that we referred to as Fluorescent Labeling in Semi-Solid Medium (FLSSM), could be used to isolate clones expressing proteins smaller than antibody molecules, which are about 150 kDa.

In the present study, we demonstrate how FLSSM is used to isolate CHO and 293SF cells secreting high-levels of recombinant proteins other than antibodies. FLSSM was compared to the traditional cell dilution in cluster wells to isolate high producers of a small protein (25 kDa) derived from the insulin like growth factor-1 (IGF-1). High producers were isolated with FLSSM more rapidly and with less effort. FLSSM was also used to isolate stable CHO cells expressing Tissue Plasminogen Activator (tPA, 67 kDa). In its simplest form, this method only requires a fluorescent antibody against the secreted product, but it can also be used with automated scanning and picking systems. FLSSM is a versatile method for the screening of high producers for research and industry.

## Results

### Isolation of clones secreting IGF-E5 using the conventional procedure

**Figure 1:**
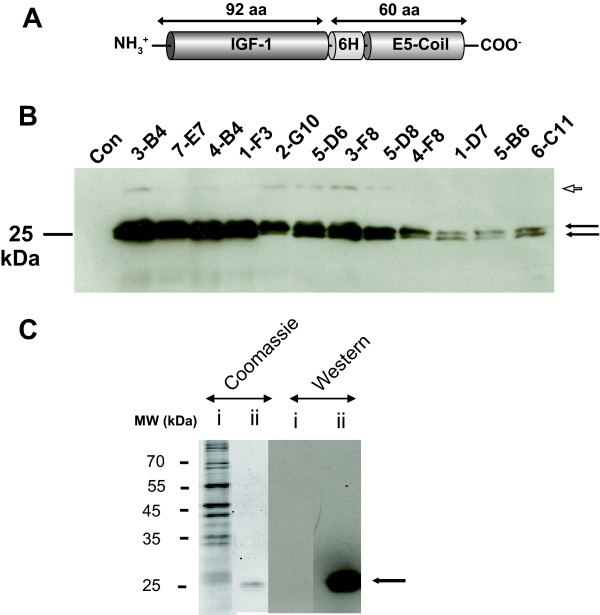
**Expression and purification of IGF-E5**. **A: **Structure of IGF-E5. This recombinant protein was made by fusing the complete amino acid sequence of the murine IGF-1 to a His Tag (6H) and to an artificial peptide referred as E5-Coil. The latter possesses a strong affinity for a complementary peptide, the K-Coil [34]. These peptides were designed to promote the interaction of protein containing E5-Coil and K-Coil with each other. The secreted IGF-E5 is 152 amino acids in length. **B: **The same amount of supernatant of clones secreting IGF-E5 after 7 days of batch culture at 30°C were analyzed by western blot using an antibody against the His Tag. The clones were obtained using the conventional procedure. Arrows: position of IGF-E5. Con: supernatant of non-transfected CHO cells. Arrowhead: position of a minor protein component of unkown nature synthetized by some clones. **C**: Purification of IGF-E5 produced by clone 4-B4 by affinity chromatography on a nickel column. Unbounded protein fraction (i) and purified IGF-E5 (ii) were analyzed by SDS-PAGE and stained using the protein dye Coomassie Fluor Orange. The same protein fractions were also analyzed by western blot using an anti-His Tag antibody. Arrow: position of IGF-E5. MW: position of molecular weight marker.

The structure of the recombinant IGF-1 protein (IGF-E5), employed to compare the conventional procedure of clone isolation (dilution in 96-well plate) with FLSSM, appears in Fig. 1A. To generate stable CHO clones secreting IGF-E5 using the conventional procedure, CHO cells adapted to protein-free suspension culture were transfected with a plasmid encoding IGF-E5 regulated by the optimized cytomegalovirus (CMV) promoter [20], as well as the hygromycin resistance gene. After transfection, the cells were separated into 96-well plates in the presence of hygromycin. Two to three weeks later, resistant cell populations from 419 wells were chosen and screened for IGF-E5 expression by dot blot. To increase the odds that the colonies originated from a single cell (a true clone), we isolated cells only from plates containing less than 30 wells with resistant colonies. Despite this precaution, some of the selected clones might have arisen from more than one cell and therefore might not be true clones. The selected populations with the highest levels of IGF-E5 were then further analyzed for IGF-E5 expression by western blot. From this experiment, equivalent cell numbers were incubated for 7 days at 30°C. We and other groups [21-24] have observed CHO cells have higher productivities at this temperature compared to the optimal growth temperature (37°C). In the following text, all experiments performed at 30°C are indicated. Equal supernatant volumes were analyzed in this western blot. Using an antibody against the His Tag, a doublet migrating around 25 kDa was detected (Fig. 1B). The same bands were also detected using an antibody against the murine IGF-1 indicating that it corresponded to the IGF-E5 (data not shown). The reason for the appearance of IGF-E5 as a doublet is not known. The 4 best cell populations were 4-B4; 3-B4, 1-F3 and 7-E7 (Fig. 1B); 4-B4 was later compared to the best clones obtained by FLSSM. For quantification, the recombinant IGF-E5 was purified by affinity chromatography on a nickel column and the amount of purified protein was determined using the Lowry assay (Fig. 1C). The purified IGF-E5 was then used to make a standard curve for quantification by spot blot (see below).

### Selection of clones expressing IGF-E5 by FLSSM

**Figure 2:**
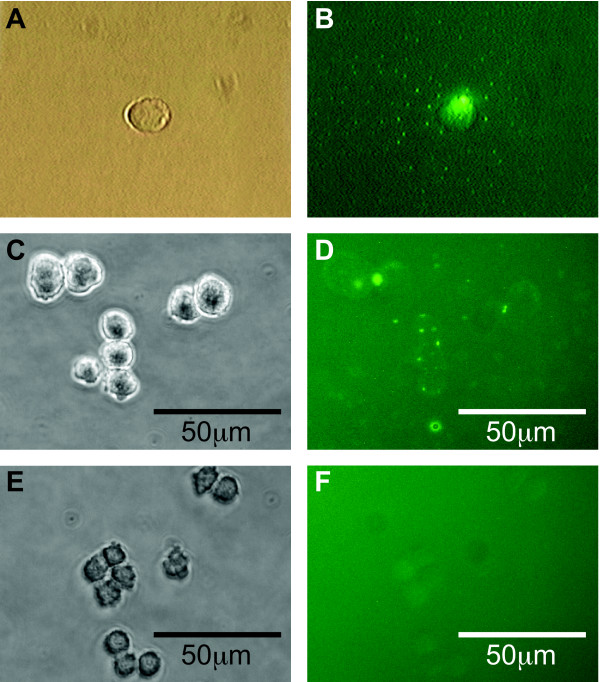
**Detection of secreted IGF-E5 from stable CHO transfectants by FLSSM**. **A**, **C **and **E**: transmitted light images. **B**, **D **and **F**: green fluorescence at 510 nm from anti-His Tag/FITC antibody present in semi-solid medium; fluorescent dots formed by reaction of anti-His Tag/FITC with secreted IGF-E5 bearing His Tag moiety. **A **and **B**: longer exposure from single cell showing a cloud of fluorescent dots. **C **and **D**: positive CHO-IGF-E5 colony: signal is weak, but sufficient for detection of high producing cells. **E **and **F**: negative control; CHO cells secreting an unrelated product do not react with anti-His Tag antibody.

**Figure 3:**
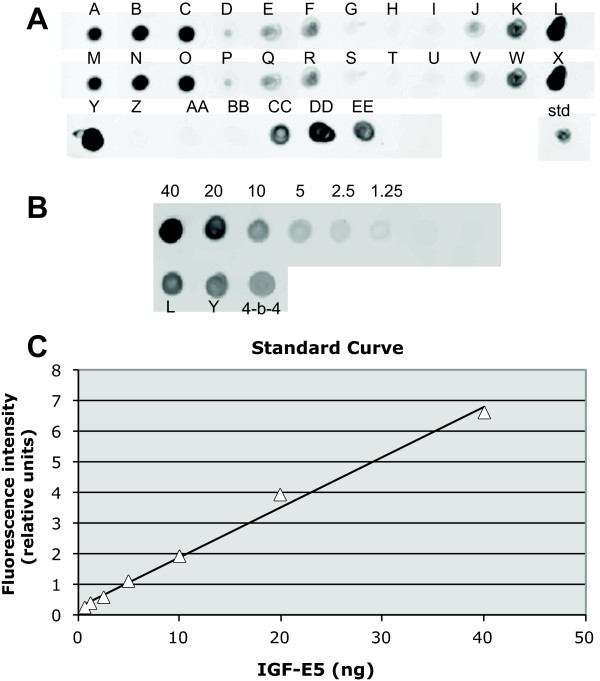
**Screening and quantification of high-producing CHO-IGF-E5 clones by spot blot**. **A**: initial screening of all colonies selected by FLSSM and picked by Quixell micromanipulator (A trough EE). std: 20 ng spot of purified IGF-E5 for reference. **B: **Quantitative spot blot. Top row: purified IGF-E5 standards, numbers indicate amounts spotted in nanograms. Bottom row: 2 best clones isolated by FLSSM (L and Y) and best cell population isolated by classic dilution in wells (4-B4). Duplicates not shown. **C**: plot of the fluorescence signal obtained by scanning the standard spots using a Typhoon™ scanner, R^2 ^= 0.99.

To test if we could isolate high producer clones more efficiently by direct labeling with a fluorescent antibody specific for the recombinant product, a pool of CHO cells expressing IGF-E5 was generated as described above. Fourteen days after electroporation and hygromycin selection, the resistant cell pool was plated in semi-solid medium containing a fluorescent anti-His Tag antibody. By microscopic observation, about 5% of cells were associated with a fluorescent signal (Fig. 2B and 2D). Control CHO cells, secreting high levels of an unrelated product, showed no fluorescent dots (Fig. 2F). From several wells of a 24-well plate, 66 single cells or small colonies bearing fluorescent dots under the microscope were picked from the semi-solid medium with a micromanipulator (Caron et al 2000) and allowed to grow in a 96-well plate without drug selection. The surviving 31 clones were first screened by spot blotting 3-day old culture supernatants. Out of 31 clones, 25 showed detectable levels of IGF-E5 (Fig. 3A).

The five clones showing the strongest signal were seeded at 5 × 10^5^cells/ml and supernatants sampled at day 3 were analyzed by quantitative spot blot. Production at 30°C from the two best clones were then compared in a final spot blot, with the starting pool as well as with the best cell population (4-B4) obtained from the conventional cloning method described above (Fig. 3B). The two best clones isolated by FLSSM secreted 307 and 276 μg/ml IGF-E5 respectively over 3 days, while 4-B4 (best by conventional procedure) secreted 267 μg/ml. The starting cell pool used for the FLSSM experiment had an IGF-E5 level below the detection limit of the blot (data not shown).

### Correlation of fluorescence intensities and secretion level

To confirm that the secreted recombinant protein levels by the CHO-IGF-E5 clones correlated well with their fluorescence intensities in semi-solid medium, we compared the IGF-E5 secretion level and the fluorescence of seven different clones under identical conditions. The clones were plated in semi-solid medium for fluorescence scanning and, in parallel, were cultured in liquid medium for a 3-day batch assay at 30°C, followed by quantitative spot blot analysis. Duplicate semi-solid medium wells for each clone were scanned with a CellCelector™ (Aviso) and the combined fluorescence for all colonies above threshold were summated. Results (Table 1) show that the seven clones ranked in the same order for production as for total fluorescence intensity, with a correlation coefficient of 0.89.

**Table 1:** Correlation between cell colony fluorescence and recombinant protein secretion from CHO-IGF-E5 clones selected by FLSSM.

Clone^a^	IGF-E5^b ^ production	SD (N = 2)	Rank^c ^ production	Total^d^ Fluorescence.	Rank^e^ Fluorescence
L	1.00	0.16	1	1.00	1
V	0.46	0.10	2	0.90	2
A	0.24	0.04	3	0.30	3
W	0.12	0.01	4	0.28	4
F	0.10	0.02	5	0.26	5
J	0.07	0.01	6	0.17	6
E	0.08	0.00	6	0.11	7

^a^Names of stable CHO clones secreting various levels of IGF-E5 (Fig. 3A).

^b^Production from a 3-day batch culture at 30°C was measured by quantitative spot blot against purified IGF-E5. The production was normalized to the highest producer (clone L).

^c^Rank for IGF-E5 production (column 2) in decreasing order. Clones J and E were both assigned rank 6, because their production level was not significantly different.

^d^Fluorescence was measured by scanning the wells at 510 nm using the CellCelector™. The fluorescence signal of all the colonies above background was summed and normalized to the highest value (clone L).

^e^Rank for the total fluorescence (column 5) in decreasing order and independently from column 4.

### CHO clones expressing tPA and 293SF expressing IGF-E5

**Figure 4:**
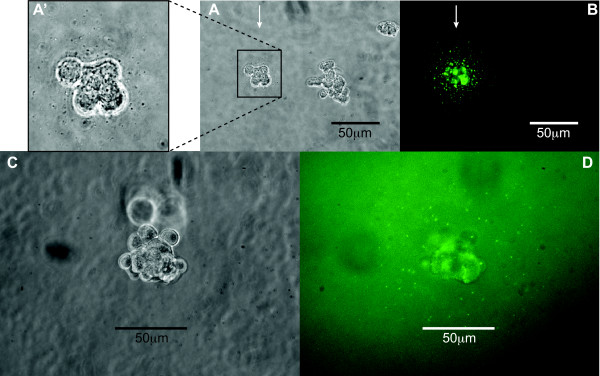
**FLSSM applied to different products and cell lines**. **A**, **A' **and **C**: transmitted light images. **A **and **B**: colonies from CHO-tPA transfectant pool. **B**: detection of secreted tPA using an anti-tPA antibody conjugated to Alexa488 in semi-solid medium. Note the presence of positive and negative colonies in the same field. Arrow indicates positive, high-producing clone; **A**': insert blown up from **A **showing that a precipitate around the positive colony is visible in transmitted light microscopy. **C **and **D**: colony from 293-IGF-E5 transfectant pool. **D**: detection of secreted IGF-E5 (small fluorescent dots at the colony periphery using an anti-His Tag antibody conjugated to FITC in semi-solid medium).

To verify if clones producing a different recombinant protein could be isolated by FLSSM, a stable pool of CHO cells expressing tPA (67 kDa), controlled by the CMV promoter, was generated. The cells were then plated at low density in semi-solid medium containing an antibody against tPA labeled with Alexa488. The fluorescent signal from positive CHO-tPA colonies was overall much stronger than that from CHO-IGF-E5 positive cells. Indeed, a precipitate around colonies was visible even in phase contrast microscopy (Fig. 4A), as is the case when the secreted product is itself an antibody [18]. From 32 positive cells and/or colonies picked, 8 showed strong growth and they were analyzed by spot blot. Five of the clones secreted more than 5 μg/ml (batch culture, 5 days, 30°C), with the best one at 9.5 μg/ml. These values are very comparable with tPA-producing cell lines generated by conventional methods in our laboratory (data not shown).

To determine if our method could be extended to other cell lines besides CHO, the human embryonic kidney 293SF cell line [25] was tested for colony formation in semi-solid medium and for detection of IGF-E5 transfectants. A stable pool of 293SF cells expressing IGF-E5 was generated and they were subsequently plated in semi-solid medium. Four days later, a liquid overlay containing the fluorescent anti-His Tag antibody was added, and the following day, the plates were scanned on a CellCelector™ automated cell picker. Despite the fact that the signal produced by the small 293-IGF-E5 colonies was relatively weak (Fig. 4B), positive clones were easily detected. Sixteen small colonies were picked and transferred by CellCelector™ to a 96-well plate. Ten of the colonies survived and they were tested by the same methods used for the CHO-IGF-E5 clones. All tested 293-IGF-E5 clones secreted significant amounts of IGF-E5. The best one produced 170 μg/ml after 5 days of batch culture at 30°C.

## Discussion

In this work we have demonstrated that FLSSM works equally well for detecting small and intermediate-sized proteins, compared to large molecules such as antibodies. We have shown that the method also worked for 293 cells. A previous study [18] and application notes [19,26] have indicated that this approach can be applied to isolate cells secreting antibodies (about 150 kDa) or membrane associated proteins. However our study is the first to demonstrate that this method can be applied to smaller soluble proteins such as IGF-E5 (25 kDa) and tPA (67 kDa). Although we confirmed that FLSSM is amenable to robotic high throughput operation using an automated cell picker such as the CellCelector™, this type of instrument is not essential to isolate high producers on a smaller scale. Indeed, this can be done as we have previously shown using a less sophisticated micromanipulator [9]. Furthermore, colonies that are big enough (>0.5 mm) and well dispersed inside a dish can be picked manually with a simple micropipettor, making this method available to virtually any laboratory.

Other methods that can isolate cell lines expressing soluble and non-fluorescent recombinant proteins have been described [5,14-17]. The challenge faced by these methods is to retain the secreted protein in the vicinity of the producer cells. This can be done using various strategies (cell biotinylation or biotinylated agarose in conjunction with avidin and biotinylated capture antibodies, or coating the plates with a capture antibody). The major advantage of FLSSM over these methods, is its simplicity, since only one fluorescent antibody is required for detection. As we have shown, FLSSM is applicable to cells that are adapted to suspension and serum free culture, the latter point being important because of safety and regulatory issues. An alternative technique used for selecting high producer clones, which consists in direct labeling of cells with a fluorescent antibody (without permeabilisation or fixation) followed by flow cytometry analysis has been described [27]. This method was tested on our strong CHO-IGF-E5 (clone L) and on a parental CHO negative control. Although we carefully followed the protocol described in this article, no significant surface IGF-E5 was detected when the antibody was used at the same concentration as for FLSSM. When we increased the cell and antibody concentrations, a signal was detected but its intensity was very weak (see additional file 1). It is not clear why cells secreting IGF-E5 are difficult to label by this method. Rapid diffusion of the molecule, due to its small size (25 kDa) and/or poor retention of IGF-E5 on the cell surface after secretion, could explain this observation. An added benefit of FLSSM is its ability to screen for growth characteristics at the same time as high protein production by analyzing the size of the colonie and the intensity of the fluorescent signal. This method can also be used to monitor clonal stability over time. A simple, regular plating in semi-solid medium will easily reveal significant drifts in homogeneity and intensity of the secreted protein amongst the progeny of an original clone.

The recombinant IGF-E5 is a good test for this method because of its relatively small size (25 kDa). We observed that the fluorescent signal with IGF-E5 was significantly weaker compared to that from tPA and an IgG (data not shown). Many factors contribute to the intensity of the detected signal: secreted product concentration, molecular size, shape, diffusion speed in methylcellulose, the detection antibody's affinity for the secreted product and the size of the colonies. The CHO-IGF-E5 positive cells were picked early after plating as single cells or very small colonies, which likely affected the survival rate of the clones. Despite this weaker signal and the very small size of the CHO-IGF-E5 colonies picked, several high CHO-IGF-E5 producers were obtained by merely analyzing a few dozen clones from FLSSM. In contrast, more than 400 cell populations had to be screened by the classic method to find a similar producer. In addition, the relative fluorescence intensity of the CHO-IGF-E5 colonies in semi-solid medium was proportional to the level of secretion of the corresponding clones in batch culture, strongly supporting the notion that high-secretors are the brighter ones. Our best CHO-tPA clone isolated by FLSSM produces 9.5 μg/ml of tPA in batch culture, which is significantly lower than our best CHO-IGF-E5. This value however is comparable to the best clones we characterized in our laboratory using the standard dilution method (data not shown). Although it is difficult to compare data obtained from different laboratories, our tPA production level is in the range of those (10 to 30 μg/ml) reported in literature for CHO cells in batch culture, without medium or growth culture optimization [28,29].

It should be pointed out that the intensity of the fluorescent signal in FLSSM should only be used as a relative index to compare different clones expressing the same product in a particular host. Case in point, the fluorescent signal surrounding CHO-tPA clones was much stronger than the signal from CHO-IGF-E5 clones, even though the best IGF-E5 clones were much higher producers. However, for a given cell host, secreted product and detection antibody, the fluorescent signal should be proportional to the level of expression, allowing isolation of the highest producers from a mixed population.

We observed some toxicity (reduced growth and lack of colony formation) when the cells were labeled with certain antibodies. Ideally, azide and thimerosal-free antibodies should be used to minimize toxicity. These and other potential contaminants present in commercial antibodies can easily be removed by microdialysis. Even though we have found that FLSSM allows detection of high producers at the single cell level, it is more advantageous and reliable to pick positive colonies of a minimal size. Accordingly, conditions favoring good colony growth should be optimized, such as medium composition, plating density and time of fluorescent antibody addition. For 293SF-IGF-E5 selection, we saw that plating cells in semi-solid medium without the detection antibody permitted initial colony growth. By adding the fluorescent label in a liquid medium overlay 3–7 days later, chances to successfully isolate high-producers improved.

Regardless of the cell selection method used, loss of expression during clone amplification is always possible if high-level recombinant protein expression is toxic and negatively affects cell growth. To that effect it would be advisable, if possible, to use an inducible, tightly-regulated promoter (such as the Tetracycline-based system [30] or the Cumate-based system [31] to drive the transgene expression. A useful strategy would be to use an inducible promoter in the "off' mode during cell colony formation, and then add the inducer to reveal the high producers for detection and picking. This should minimize negative selection and favor stability of the high-secretor clones. It has been shown that secreted product detection is possible even with an un-labeled antibody against the product [18]. Indeed, we have found that the precipitate formed by the secreted product and the detection antibody around high-secretors is readily visible in phase contrast or dark field microscopy.

## Conclusion

Fluorescence labeling in semi-solid medium (FLSSM) allows rapid visualization of the high secretors from transfected cell pools prior to picking, thus eliminating the tedious task of screening a high number of cell isolates. It can be used to select for growth characteristics at the same time as high protein production. FLSSM should be applicable for the isolation of most secreted protein producers, provided that the product is secreted at a sufficient level and that an antibody against it is available. It is far less labor intensive than traditional methods and represents large savings in time, supplies and incubator space. In addition, the method has been tested successfully on micromanipulator and robotic systems.

## Methods

### Plasmid construction

Restriction and DNA modifying enzymes were purchased from New England Biolabs, Ltd (Pickering, Ont, Canada). Plasmids were constructed using standard molecular biology procedures and they were purified with the Qiagen plasmid Maxi Kit (Qiagen, Valencia, CA). To construct pMPG-IGF-E5, the coding sequence of IGF-1 was isolated by PCR from the murine cDNA of that gene (GenBank ID: BC012409, BF383724, ATCC, Manassas VA,) and primers 5'-ggatccggcaccatgtcgtcttcacacctc-3' and 5-ggtacctccacctgaaccggctgcttttgtaggcttc-3'. The PCR product was phosphorylated by treatment with T4 polynucleotide kinase and cloned into the unique *PmeI *site downstream of the CMV promoter of pMPGB34k, which was previously digested with *BamHI *to remove the light chain of the B43 antibody [32]. pMPGB43k is an expression vector that carries an enhanced version of the human CMV enhancer/promoter [20] and a hygromycin resistance gene. The DNA fragment encoding the His Tag and E5-Coil sequence [33,34] was amplified by PCR using primers 5'-tattggtaccggcgggcaccatc-3' and 5'-gcgaggtaccgctagcttattacttctcaagtgctg-3'. The PCR product was digested with *Kpn*I and cloned into the unique *Kpn*I site downstream of the IGF-1 sequence previously inserted into pMPGB43k. The complete nucleotide sequence of IGF-E5 was confirmed by DNA sequencing. Plasmid pMPG-tPA, encoding tissue plasminogen (tPA) regulated by the CMV promoter and hygromycin resistance, was constructed by isolating the tPA cDNA by PCR from pETPFR (American Type Culture Collection). A first amplification was performed using primers AS1: 5'-gccgccaccatggatgcaatgaagaga-3' and AAS1: 5'-gttggatcctcacggtcgcatgttgtc-3' which inserted a Kozak consensus sequence at the 5' end and a unique *BamH*1 at the 3'end. A *BamH*I site was then added at the 5' end by performing a second PCR reaction with primers AS2: 5'-aacggatccgccgccaccatggatgca-3' and AAS1. The light chain of B43 antibody from pMPGB43k was then replaced with the PCR product by digestion with *BamH*I.

### Cell culture

CHO cells adapted to serum-free suspension culture (CHO-SF, Invitrogen, Grand Island, NY), were grown in protein free chemically defined medium for CHO cells (CD-CHO medium, Invitrogen) supplemented with 1× HT supplement (Invitrogen), 4 mM glutamine and 50 μg/ml dextran sulfate (MW: 500 000, Amersham Pharmacia Biotech, Uppsala, Sweden). 293SF [25] were grown in low-calcium-SFM (LC-SFM, Invitrogen, Grand Island, NY) supplemented with 1% fetal bovine serum (HyClone, Logan, Utah). Unless mentioned otherwise, all the cells were grown at 37°C in the presence of 5% CO_2_.

### Transfection and stable clone production

CHO-SF and 293SF were transfected by electroporation in the presence of linearized plasmid using a BTX T820 electrosquare porator (Genetronics, San Diego, CA) according to the manufacturer's recommendations. For the clone isolation using the "standard method", after electroporation, the CHO-SF were diluted into growth medium and incubated overnight in 6-well plates. The next day, they were separated into 96-well plates (1000 and 3000 cells/wells) in growth medium supplemented with 600 μg/ml hygromycin B (Invitrogen). Resistant colonies, isolated from plates in which less than 30 wells contained resistant colonies to maximize the odds of isolating a true clone, were then transferred to 24-well plates in the presence of hygromycin B. Supernatant from confluent 24-well plates were analyzed by dot blot for the presence of IGF-E5. To generate clones using the FLSSM method (see below), after electroporation, a pool of stable transfectants was generated by growing the cells in the presence of 600 μg/ml (CHO-SF) or 25 μg/ml (293SF) hygromycin B.

### Cell plating in semi-solid medium

Immediately before plating in semi-solid medium, cells were dispersed in a 35 mm Petri dish through a fine microtip (Costar, Bethesda, MD) and observed under the microscope. The process was repeated until only single cells were observed. CHO-IGF-E5 and CHO-tPA stable cell pools were mixed in medium containing Clonematrix methylcellulose, XL Reagent (Genetix, Boston, MA) and CDCHO-AGT 2× (Invitrogen), at a 1× final concentration and supplemented with 8 mM L-glutamine (Invitrogen) and fluorescent antibody (see below). 293SF-IGF-E5 stable cell pools were mixed in medium containing Clonematrix, 2× LC-SFM medium (1× final concentration), 1%FBS (HyClone) and 4 mM L-glutamine. The cells (CHO and 29SF) were agitated and vortexed at medium speed to ensure uniform distribution of all components before plating. They were then seeded in 24-well dishes (Sarstedt, Newton, NC) or 60 mm Petri dishes (Greiner, Monroe, NC), made of untreated plastic, at densities of 500, 1000 and 2000 cells/m. After plating, the cells were observed under the microscope to verify they were single and well dispersed inside the dish.

### Cell labeling and colony picking

Anti-His Tag-FITC antibody (Abcam, Cambridge, MA) was used at 4 μg/ml to detect IGF-E5 secreting cells in semi-solid medium. The anti-tPA (Cedarlane, Hornby, Ont) antibody was first dialyzed on a microcon YM-50 spin filter (BioRad, Missisauga, ON) against PBS (HyClone), and conjugated to Alexa488 fluorochrome with a Microscale Protein Labeling Kit (Molecular Probes, Eugene OR) according to the manufacturer's recommendations. The labeled antibody was used at a concentration of 1 μg/ml to detect tPA-secreting cells. Positive cells and colonies were picked either with a Quixell micromanipulator (Stoelting, Wood Dale, IL) using 50 μm glass capillaries, or with a CellCelector™ colony picker (Aviso, Jena, Germany) equipped with the methylcellulose tool. For Quixell picking, the most fluorescent cells were determined visually. For CellCelector picking, the plates were scanned at 10× magnification and the brightest objects were determined using the included analySIS software (now called Cell D). Single cells and colonies were deposited into 96-well dishes (Corning, Lowell, MA). Images of fluorescently labeled cells were taken on a Leica DMIL inverted microscope using a color 3CCD cooled camera (Optronics, Goleta, CA) or a monochrome Retiga Exi (QImaging, Surrey, BC) and Openlab software (Improvision, Coventry, England).

### Analysis of protein production by dot blots and spot blots

For dot blot analysis, the culture supernatant was diluted 5 times with distilled water. The samples were denatured by adding an equal volume of 2× lysis buffer (0.125 M Tris-HCl pH 6.8, 4% SDS, 20% glycerol and 0.1% bromophenol blue) and heated at 95°C for 5 min. The samples were spotted on a nitrocellulose membrane (Hybond-ECL, Amersham Biosciences Buckinghamshire, UK) under vacuum on a dot-blot apparatus (Bio-Rad Laboratories Canada Ltd., Mississauga, ON). The membrane was incubated with an anti-His Tag antibody (Serotec, Oxford, UK) followed by a horseradish peroxidase-conjugated anti-mouse IgG antibody (Amersham Biosciences). The signal was revealed by chemiluminescence using the ECL Western Blotting detection reagents (Amersham Biosciences). For spot blot analysis [35] duplicate or triplicate volumes of one microliter were spotted on nitrocellulose membranes (Hybond-ECL) with a precision P2 micropipette (Eppendorf, New York, NY). Quantitative spot blots were made from batch samples, from clones seeded at 5 × 10^5 ^cells/ml and grown for 3 days (CHO-SF) or 5 days (293SF) under identical conditions at 30°C. At least three dilutions were spotted for each clone to ensure that detected values would fall in the linear domain of the standard curve. IGF-E5 blots were treated with an anti-His Tag antibody (Serotec) followed by an anti-mouse conjugated to Alexa488 (Invitrogen). tPA blots were treated with a sheep anti-tPA primary antibody (Cedarlane) followed by a rabbit anti-sheep antibody conjugated to Cy3 (Jackson, Immunoresearch Labs, West Grove, PA). Protein standards (purified IGF-E5 or tPA (Calbiochem, San Diego, CA)) were spotted in duplicate or triplicate serial dilutions on each blot. The blots were scanned on a Typhoon Trio+ fluorescence imager (Amersham Biosciences) and analyzed by ImageQuant TL software (Amersham Biosciences). The data was transferred to Excel spreadsheets for standard curve plotting and result interpolation.

### Analysis of protein production by Western blot

Stable CHO transfectants (5.0 × 10^5^) were resuspended into 1 ml of growth medium in 24-well plates. The next day, the plates were transferred to an incubator at 30°C and incubated at this temperature for up to 14 days. At selected time points, an aliquot of growth medium was mixed with an equal volume of lysis buffer 2× supplemented with 5% β-mercaptoethanol. The samples were heated for 5 min at 100°C and separated on a SDS-PAGE. The proteins were transferred onto a nitrocellulose membrane and the presence of the IGF-E5 was demonstrated using anti-His Tag antibody by chemiluminescence as described above.

### Purification of IGF-E5

Actively growing CHO cells from clone 4-B4 (1.0 × 10^6^/ml) in a T-Flask were incubated at 30°C for 7 days. After separation of supernatant and cells by centrifugation, Complete protease inhibitor (Roche Diagnostics, Mannheim, Germany) and a final concentration of 10 mM imidazole were added to the supernatant. The supernatant was loaded onto a 5-ml HisTrap column (Amersham Biosciences) using a peristaltic pump and the bound IGF-E5 was eluted with increasing concentrations of imidazole (10 to 200 mM). The purified IGF-E5 was dialyzed against PBS and concentrated on a centrifugal concentrator (Centricon YM-10, Millipore Corporation, Bedford, MA). After determining the protein concentration with the DC protein assay (Bio-Rad laboratories, Hercules, CA), the purified IGF-E5 was stored at -80°C in 5% glycerol. The purity of the protein was evaluated by SDS-PAGE followed by staining with Coomassie Fluor™ Orange protein gel stain (Molecular Probes, Eugene OR) and analyzed on a Typhoon trio+ scanner (Amersham Biosciences). The nature of the purified IGF-E5 was confirmed by Western blot (as described above) using as primary antibody an anti-His Tag antibody or a goat anti-IGF-1 antibody (USBiological, Swampscott, MA).

### Correlation of production and fluorescence in FLSSM

Seven CHO-IGF-E5 clones of various production levels (as per initial spot blot screening) were seeded at 1.0 × 10^4 ^cells/ml in semi-solid medium containing anti His Tag-FITC antibody in duplicate wells of a PetriWell24 plate (Genetix) and, in parallel batches at 1.0 × 10^6 ^cells/ml in liquid growth medium. Duplicate wells of a 24-well dish (Sarstedt, Montréal, QC) were incubated at 30°C for 3 days. Samples were clarified by centrifugation and analyzed by quantitative spot blot as described above. Semi-solid medium plates were scanned on a CellCelector™ (Aviso). The product of area and mean fluorescence for all the colonies above background threshold was determined with the Cell D software (Aviso/Olympus) and summed for each separate clone using an Excel spreadsheet. The results were normalized to the brighest clone (L). Quantitative spot blot results for batch production were normalized to the highest producer (L).

## Authors' contributions

AC developed the FLSSM method, did the microscopic analysis, generated clones, quantified their expression and drafted the manuscript. CN generated the IGF-1 expressing clones and quantified their expression, BG produced the tPA expressing plasmid, developed the spot blot quantification and quantified the CHO-tPA clones. IB constructed the IGF-1 ligand, MP produced the IGF-1 ligand and developed a purification method for it. AG supervised the work of BG and participated in the design of tPA production and quantification, BM provided the original idea for the study and interpreted the data, RG conceived of the study, participated in its design and co-drafted the manuscript. All authors read and approved the final manuscript.

## Supplementary Material

Additional File 1 **Identification of high-secreting CHO clones: comparison between surface detection by flow cytometry and FLSSM**. Additional data showing labeling of CHO-IGF-E5 cells with the anti His Tag-FITC antibody followed by flow cytometry. Click here for file
