# Supplementary material for: Fluorescent labeling in semi-solid medium for selection of mammalian cells secreting high-levels of recombinant proteins
Source: BMC Biotechnol. 2009 May 11;9:42. doi: 10.1186/1472-6750-9-42 (PMC2689207; doi:10.1186/1472-6750-9-42)
Supplement: Additional File 1 — Identification of high-secreting CHO clones: comparison between surface detection by flow cytometry and FLSSM. Additional data showing labeling of CHO-IGF-E5 cells with the anti His Tag-FITC antibody followed by flow cytometry. [file 1472-6750-9-42-S1.pdf]

IDENTIFICATION OF HIGH-SECRETING CHO CLONES:
Comparison between surface labeling detection by Flow Cytometry and FLSSM
(Fluorescent Labeling in Semi-Solid Medium)

A

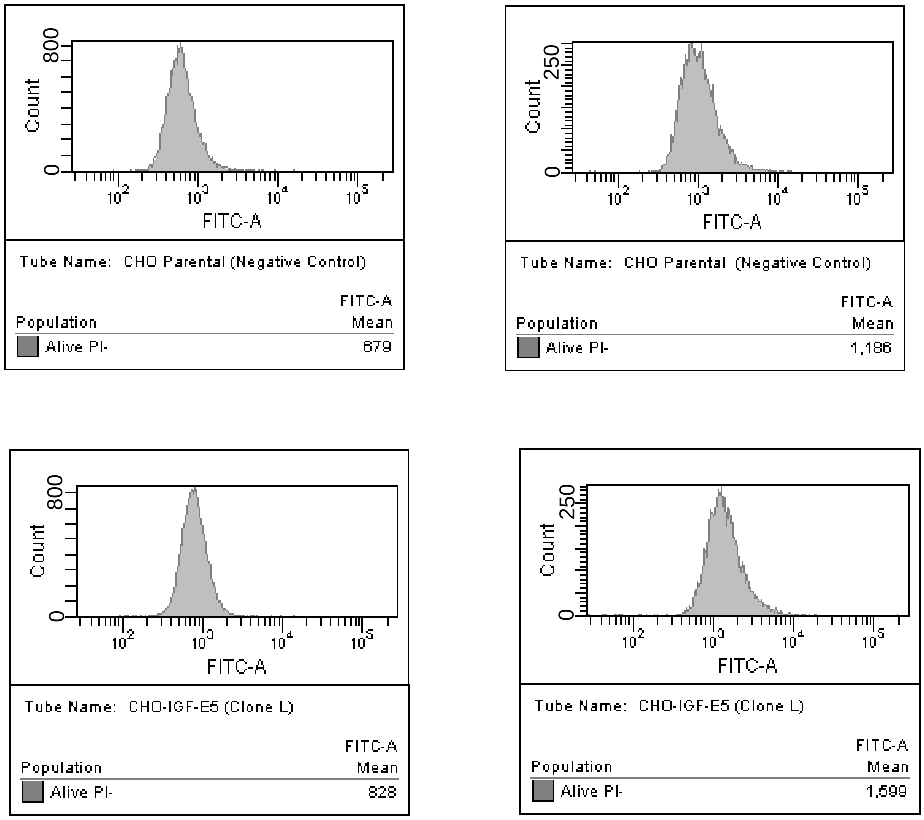

CHO parental negative control

CHO-IGF-E5 Clone “L”

HiVolt 450

HiVolt 450
Cell Conc x2, Ab Conc x3

Note: gate is on propidium iodide-negative (live) cells

A: Cell surface measurement of IGF-E5 by flow cytometry.
CHO-IGF-E5 (clone L) and CHO parental cells were labeled with an anti His tag-FITC antibody as described by Brezinsky et al (27). The cells were then analyzed by flow cytometry. The antibody was used at the same concentration as for FLSSM or three times more concentrated. A weak signal was detected only at higher antibody concentration

B

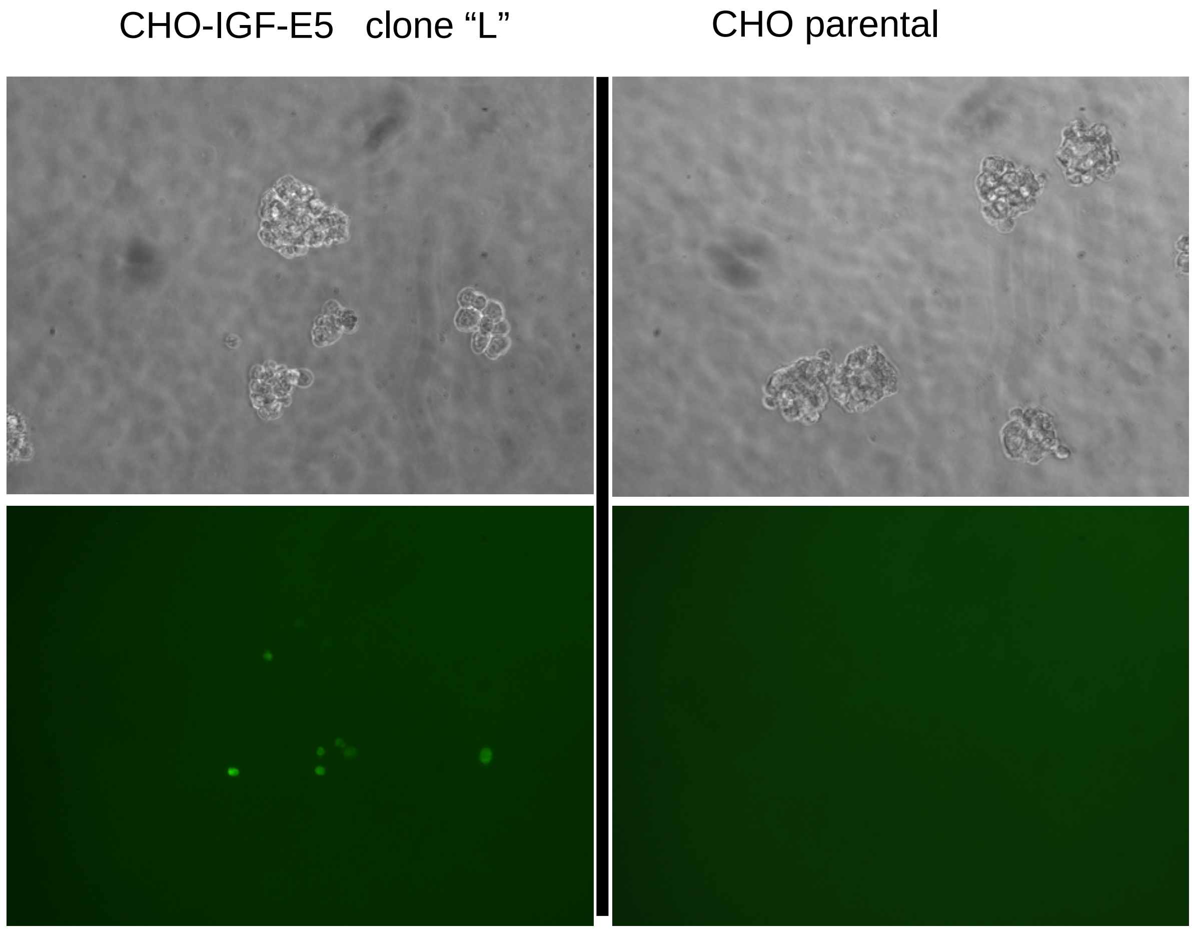

B: Fluorescent Labeling in Semi-Solid Medium
Cells processed in parallel for FLSSM using the anti His tag-FITC antibody. Images captured with a cooled CCD camera. Note the presence of fluorescent dots at the periphery of the CHO-IGF-E5 cells, but not at the periphery of parental cells.
